# Biological Significance of the Genomic Variation and Structural Dynamics of SARS-CoV-2 B.1.617

**DOI:** 10.3389/fmicb.2021.750725

**Published:** 2021-10-07

**Authors:** Lin-qian Fan, Xiao-yun Hu, Yi-yue Chen, Xiang-lei Peng, Yuan-hui Fu, Yan-peng Zheng, Jie-mei Yu, Jin-sheng He

**Affiliations:** College of Life Sciences and Bioengineering, Beijing Jiaotong University, Beijing, China

**Keywords:** SARS-CoV-2, B.1.617, genetic diversity, molecular evolution, haplotype network, tertiary structure

## Abstract

Severe acute respiratory syndrome coronavirus 2 (SARS-CoV-2) variants have been emerging and circulating globally since the start of the COVID-19 pandemic, of which B.1.617 lineage that was first reported in India at the end of 2020, soon became predominant. Tracing genomic variations and understanding their impact on the viral properties are the foundations for the vaccine and drug development and for the mitigation measures to be taken or lifted. In this study, 1,051 near-complete genomes and 1,559 spike (S) sequences belonging to the B.1.617 were analyzed. A genome-wide spread of single nucleotide polymorphisms (SNPs) was identified. Of the high frequency mutations identified, 61% (11/18) involved structural proteins, despite two third of the viral genome encoding nonstructural proteins. There were 22 positive selection sites, mostly distributed across the S protein, of which 16 were led by non-C to U transition and should be of a special attention. Haplotype network revealed that a large number of daughter haplotypes were continually derived throughout the pandemic, of which H177, H181 H219 and H286 from the ancestor haplotype H176 of B.1.617.2 were widely prevalent. Besides the well known substitutions of L452R, P681R and deletions of E156 and F157, as well as the potential biological significance, structural analysis in this study still indicated that new amino acid changes in B.1.617, such as E484Q and N501Y, had reshaped the viral bonding network, and increasingly sequenced N501Y mutant with a potential enhanced binding ability was detected in many other countries in the follow-up monitoring. Although we can’t conclude the properties of all the mutants including N501Y thoroughly, it merits focusing on their spread epidemically and biologically.

## Introduction

The COVID-19 pandemic caused by the severe acute respiratory syndrome coronavirus 2 (SARS-CoV-2) is spreading globally. As of June 15, 2021, more than 175 million cases and nearly 3.9 million deaths have been confirmed^1^. Coronaviruses are enveloped, positive-sense RNA viruses that undergo rapid mutation and recombination (Zhao et al., 2004; Su et al., 2016). As expected, the spread of SARS-CoV-2 has generated a large number of variants. To this end, a variant classification scheme has been developed to assign the major variants to one of three classes: variant of interest (VOI), variant of concern (VOC), and variant of high consequences (VOHCs). To date, no VOHCs have been determined, while four variants have been considered as VOI (B.1.525, B.1.526, B.1.617.1, and C.37) and another four as VOC (B.1.1.7, B.1.351, B.1.617.2, and P.1). Of note, the variants in each of the three classes would be constantly updated upon continual surveillance of the risks to global public health.

The B.1.617 lineage of SARS-CoV-2 was first reported in India in October 2020 and has spread to numerous countries and regions. As of June 15, 2021, three B.1.617 sublineages with distinct mutations have emerged: VOI B.1.617.1 (Kappa), VOC B.1.617.2 (Delta), and VOI B.1.617.3. Though the amino acid (aa) changes of G142D, L452R, D614G, and P681R occurring in the spike (S) protein are signatures for B.1.617 and present in all the three sublineages, distinct mutation profiles were found in each of the sublineages. For example, T478K aa change in the receptor-binding domain (RBD) and 156–157 deletions in the N-terminal domain (NTD) of the S protein are unique to B.1.617.2, while E154K and Q1071H substitutions exist only in the B.1.617.1 sublineage. The mutation features warrant further assessment both individually and as a whole for the B.1.617 sublineages. Some of the aa changes or similar ones in B.1.617 were also identified in other circulating lineages: D614G was also found in B.1 lineage, L452R in B.1.526 (Iota), and P681H in B.1.1.7 (Alpha). L452R is located in the RBD, and P681H or P681R is located in the furin cleavage site. It was observed that the variants carrying spike L452R change are likely to be more transmissible and infective and less susceptible to the neutralizing antibodies from convalescent patients and vaccine recipients (Deng et al., 2021). The variant bearing P681R, such as B.1.617.2, has an increased furin-mediated cleavage at the S1/S2 cleavage site that would lead to enhanced viral fusogenicity and exhibit a higher pathogenicity (Liu et al., 2021b; Peacock et al., 2021; Saito et al., 2021). It was also observed that in the hamster model, B.1.617.1 has a higher pathogenicity than has B.1 (Yadav et al., 2021a). Moreover, it has been reported that B.1.617 variant has a reduced neutralization than the Wuhan prototype and B.1.1.7 by using monoclonal antibodies and/or convalescent sera from infected and vaccinated individuals (Hu et al., 2021; Liu et al., 2021a; Planas et al., 2021; Yadav et al., 2021b).

The understanding of sequence changes is important in clarifying the impact of different residues on the viral properties, such as transmissibility, pathogenicity, infectivity, and immune escape potential. Since only a short time has elapsed since the emergence of the B.1.617 variants, limited evidence has been available to establish the relationship between the aa differences and the phenotypical or epidemiological impact of each emerging variant. Thus, a close surveillance on viral genomic variation and analysis of the changes on the potential biological significance is needed. In this study, we examined the genomic signatures, spatial–temporal dynamics, and tertiary structures of functional proteins bearing the aa changes in B.1.617 viruses. Our results exposed a large number of mutations distributed across the B.1.617 viral genomes. Novel haplotypes have evolved from the original ones, some of which are widely prevalent. Evidence of selective pressure was detected at specific sites on viral protein-coding genes. Specifically, aa substitutions at positions 484 and 501 in the RBD appeared to alter the protein tertiary structure and may affect the biological activity of the B.1.617 variants. This study would help us thoroughly understand the genomic and phenotypic features of the B.1.617 variants and provide a foundation to explore novel strategies for the development of vaccines and anti-viral drugs.

## Materials and Methods

### Retrieval and Selection of Viral Sequences

SARS-CoV-2 genomic sequences were gathered from the Global Initiative on Sharing All Influenza Data (GISAID^2^) by selecting the “VOC G/452R.V3 (B.1.617.+)” as the “Variants” search term. The sequences were downloaded, ensuring that no more than five sequences were collected from the same day and the same country or region. Low-quality full-length genome and S-encoding gene (with gap or ‘‘Ns’’) sequences were removed. The Wuhan-Hu-1/2019 prototype genomic sequence (NC_045512.2) was downloaded from the National Center for Biotechnology Information (NCBI) GenBank Database^3^ and used as reference for mutation site and structure annotation.

### Calculation of Genetic Diversity

All the sequences were annotated by the accession number, collection date, and location and were aligned using the MAFFT version 7 multiple sequence alignment program (online version^4^). The number of single-nucleotide polymorphisms (SNPs) across the SARS-CoV-2 genomes was analyzed online^5^ in the *Coronaviridae* taxon. The relative frequencies of aa occurrence (bits) at informative sites were visualized using the online version of the WebLogo sequence logo generator^6^.

### Selective Pressure Analysis

In order to characterize the B.1.617 variations and to localize statistically supported positively and negatively selected sites, the selective pressure analysis was performed using the Datamonkey adaptive evolution server (online version^7^) (Pond et al., 2012; Spielman et al., 2019). Positive (or diversifying) selection is defined as sites statistically significant for a positive value of non-synonymous (dN) to synonymous (dS) substitution ω > 1, negative (or purifying) selection is inferred for ω < 1, and neutrality is ω = 1 (Zhang et al., 2005). To reduce the impact of high possibility of occasional events on the analysis based on the microevolution scale, three site-level selection methods—mixed-effects model of evolution (MEME), fast unbiased Bayesian approximation (FUBAR), and single-likelihood ancestor counting (SLAC)—were applied, and their results were merged in this study. MEME reports positively selected sites that consider both pervasive and episodic selection using a mixed-effects maximum likelihood approach; it infers an α (dS) value and two β (dN) values (β– and β+) per-site with two ω rates fitting a null and alternative model each; a site is under the positive selection if β+ > α and shown to be assessed significantly by the likelihood ratio test; FUBAR is a method for detecting site selection on datasets including more than 500 sequences; it uses a Bayesian approach to infer dN and dS substitution rates on a per-site basis and reports evidence of selections using posterior probabilities; it finds sites in which positive selection is working in case of posterior probabilities ≥0.9; SLAC is a counting-based method that uses maximum likelihood inferring ancestral characters for individual site across the phylogeny; it directly counts the number of synonymous and non-synonymous changes at each site over evolutionary time, and positive selection is defined if non-synonymous changes are more than those in the site under neutrality (Spielman et al., 2019).

#### Haplotype Network Performance

DnaSP v5.0 was used to define sequence sets and generate multi-sequence-aligned haplotype data in the rdf file format. Phylogenetic network analyses were performed using the Network 10200 package, based on the haplotypes generated by DnaSP. The data were run with the epsilon parameter set to zero, and an exploratory run was performed by setting the epsilon parameter to 10 with the median joining network algorithm. The network output was annotated using the Draw Network option to indicate geographical and time distribution, as well as cluster nomenclature.

#### Reconstruction of Geographical- and Time-Scaled Phylogeny

The aligned S gene sequences were used in the phylogenetic analysis. The phylogenetic tree was generated using MEGA 7.0.26 software, employing the maximum likelihood method with the Kimura 2-parameter substitution model.

#### Spatial Structure Construction and Analysis

The tertiary structures of the B.1.617 variant S protein RBD mutants were predicted using the Phyre2 server^8^ (van Dorp et al., 2020). Protein--protein interactions were examined using the HDOCK server^9^, based on a hybrid algorithm of template-based modeling with default parameters (Islam et al., 2020). The structures were calculated and visualized using PyMOL software version 2.

## Results

### Sequence Information

**FIGURE 1:**
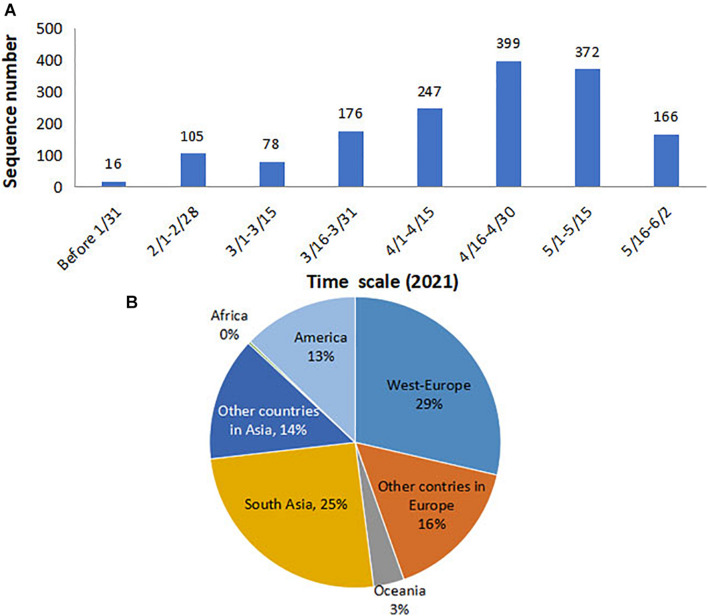
Time and location distributions of the SARS-CoV-2 B.1.617 sequences collected for the purpose of this study. **(A)** Monthly distribution: the number of sequences increased sharply since March 2021. **(B)** Geographical distribution: 29 and 25% of sequences were from Western Europe and Southern Asia, respectively. SARS-CoV-2, severe acute respiratory syndrome coronavirus 2.

The B.1.617 sequences analyzed in this study were collected between December 1, 2020, and June 2, 2021. A total of 1,051 high-quality, near-complete genomes and 1,559 S gene sequences were retrieved (Supplementary Material). As the spatial–temporal analysis in this study was performed based on the S gene, we calculated the collection time and location for the 1,559 sequences. The data showed that the sequences included in the present study covered each month of the 6-month B.1.617 pandemic, during which the sequence number prior to February 2021 was relatively small and then increased from March 2021 (Figure 1A). With regard to geographic distribution, 29 and 25% of sequences originated from Western Europe and Southern Asia, respectively, while only ∼3% of the sequences were derived from Oceania and Africa (Figure 1B).

### High Genetic Diversity and Amino Acid Changes Occurring in B.1.617 Lineage

**FIGURE 2:**
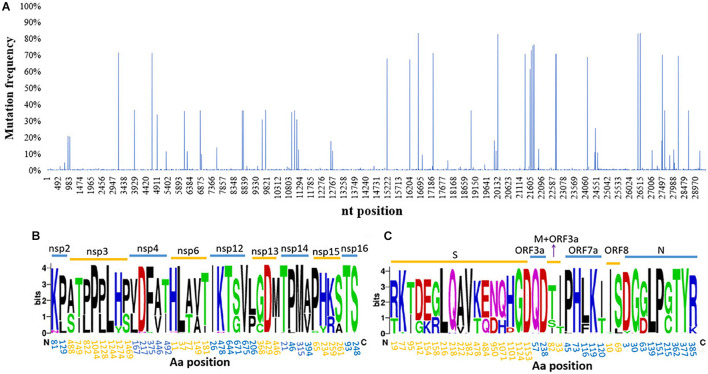
Genetic diversity and amino acid (aa) substitutions affecting SARS-CoV-2 B.1.617. **(A)** The distribution of SNPs; a large number of SNPs were found widely distributed throughout the viral genomes sampled. Informative aa sequences detected in **(B)** non-structural proteins and **(C)** structural proteins. Informative sites were defined as locations where >1% of the sequences had a different aa at that position. The names of different proteins are indicated above the line and aa positions within each protein below the line. SARS-CoV-2, severe acute respiratory syndrome coronavirus 2; SNP, single-nucleotide polymorphism.

The total nucleotide (nt) identity of the analyzed 1,051 near-complete genome sequences was 99.9%. However, when comparing the sequences to the early B.1.617 sequence identified in December 2020 (EPI_ISL_1360317), 1,857 nt sites with a mutation frequency >1% were found across the genome (29,424 nt). A higher occurrence frequency in the coding genes of structural protein than non-structural proteins was observed (Figure 2A). Eighteen nt sites with mutation frequency >60% were identified, of which 11 were located in the structural protein-coding regions and eight within the S gene (C56G, T284C, C333T, G425A, A460G, C1433A, C1450G, and G2848A). Of these eight mutated sites, seven were non-synonymous mutations (T19R, I95T, G142D, K154E, T478K, Q484E, and D950N), and one was a synonymous mutation (D333).

A total of 76 aa substitutions with an occurrence frequency >1% were found in B.1.617, of which 39 were positioned in non-structural proteins and 37 in structural proteins. When the proteins were further analyzed individually, the S protein was found to contain the largest number of substitutions, followed by the nsp3 and nsp12 proteins. There were no substitutions in the nsp1, nsp7-11, E, or ORF10 proteins (Figures 2B,C). More than a quarter (27.6%, 21/76 sites) of all the substitutions targeted the S protein, of which 11 had high occurrence frequencies (>10%) and three (aa 382, 478, and 484) were located in the RBD. Additionally, aa deletions were also found in the S (E156Δ and F157Δ), nsp1 (K142Δ, S143Δ, and F144Δ), and ORF8 (D119Δ and F120Δ) proteins with 69.5, 44.3, and 1.1% occurrence frequencies, respectively. The RNA-dependent RNA polymerase (RdRp), nsp12, contained 5 aa substitutions with an occurrence frequency of over 1%: T26I, K478N, T643I, G671S, and V675I, of which G671S had the highest frequency (67.8%) (Figure 2B).

### Specific Sites Under Selective Pressure

Diversifying and purifying selection sites on the B.1.617 coding sequences were estimated. The sites that were supported by at least two methods (*p*-value < 0.1 in MEME and SLAC or posterior probability >0.9 in FUBAR) were considered as candidates under selective pressure, and the sites supported by all the three methods were defined as strong selection sites. Results showed that the selection pressure varied markedly among different proteins. Neither positive nor negative selection sites were found in the nsp1, nsp4, nsp5, nsp8, nsp11, E, ORF3a, ORF6, ORF7b, ORF8, or ORF10 coding sequences. In contrast, both positively and negatively selected sites were identified in the nsp6, nsp12, nsp13, nsp16, S, and nucleocapsid (N) coding sequences. A total of 29 negatively selected sites were found in 11 different proteins, of which 22 (75.9%) were located in the non-structural proteins. Meanwhile 22 positively selected sites were found in nine different proteins, with 14 (63.6%) sites distributed in the structural proteins, of which nine (41%) were located in the S protein. Additionally, six of these 22 sites were led by C-to-U transition (R134N in nsp10, A394V in nsp14, A222V and H1101D/Y in S, and P13T and T135I in N). As C-to-U transition is a preferred direction of nucleotide mutations in SARS-CoV-2 (Matyasek and Kovarik, 2020; van Dorp et al., 2020), we should pay more attention on the 16 positive selection sites that were mutations of other directions than C to U. Notably, seven sites, of which four (57%) were located in the S protein, were identified under strong positive selection (Table 1). No positive selection sites were found localized in the RBD.

**TABLE 1:** Positively and negatively selected sites in coding regions of B.1.617.

**Protein name**	**Positively selected sites**	**Negatively selected sites**
nsp2	–	T161, Y207
nsp3	–	D174, Y246, T955, C1029, Y1055, F1107, F1142, K1482, A1739
nsp6	**L37F**	F34, F216
nsp7	D77G	–
nsp10	R134N	–
nsp12	S647I, **G671S**	V71, H99, D274, K417, I450, F766
nsp13	C206G	N268
nsp14	A394V	–
nsp15	–	V121
nsp16	R216N	G155
S	**I95T**, **G142D**, **K154E**, A222V, R452L, S943P, **Q1071H**, H1101D/Y, V1264L	D111, V1061, Y1215
M	–	N41
N	D3Y, P13T, **D63G**, T135I, G215C	H145, F274
ORF7a	–	Y40

*“–”: with no selective site. Sites in bold indicated a strong positive selection. Coding regions with no positive and negative sites are not listed in the table.*

### Spatial–Temporal Distribution of B.1.617 Sublineages

Among the 24 mutated nucleotide sites with occurrence frequency of more than 1% in the 1,559 S gene, 13 were transitional mutations and 11 were transversional ones. The C-to-U transition (six sites) and G-to-U transversion (five sites) accounts for 45.8% of the mutations, while the rest of the 10 mutation directions account for the 54.2%. The overall estimated bias of transitions and transversions in the selected lineage is 5.23.

**FIGURE 3:**
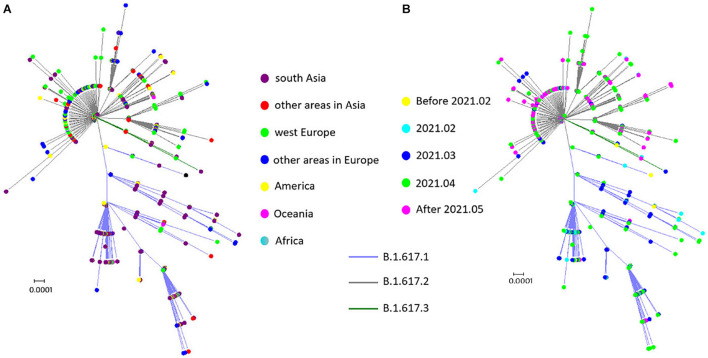
Spatial–temporal distribution of sublineages in B.1.617. **(A)** Spatial distribution: no spatial specificity was found for the B.1.617.3 sublineage, B.1.617.1 was mainly localized to Southern Asia, and B.1.617.2 was distributed worldwide. **(B)** Temporal distribution: B.1.617.1 and B.1.617.3 were prevalent in the early stages of the pandemic, while B.1.617.2 was the predominant sublineage after March 2021.

To trace the history of potential B.1.617 sublineage spread, an unrooted maximum likelihood tree was built using the 1,559 S protein encoding sequences. B.1.617.3, the first reported sublineage, was less prevalent at the time of analysis; and therefore a relatively small number of sequences was included in this study, which did not display any obvious spatial or temporal specificity (Figure 3). Apparent temporal specificity was found in both B.1.617.1 and B.1.617.2 sublineages. Initially (between the end of 2020 and March 2021), B.1.617.1 was more prevalent. Then, in April 2021, the prevalence of B.1.617.1 and B.1.617.2 became equivalent. Finally, in May 2021, B.1.617.2 overtook B.1.617.1 to become the dominant sublineage (Figure 3B). Moreover, with regard to geographical distribution, B.1.617.1 was mainly localized to Southern Asia, while B.1.617.2 acquired worldwide distribution (Figure 3A). The temporal and geographical specificity was caused by the fact that since its emergence, B.1.617.2 quickly replaced B.1.617.1 as the main prevalent sublineage since its emergence.

### Haplotype Network Generation

**FIGURE 4:**
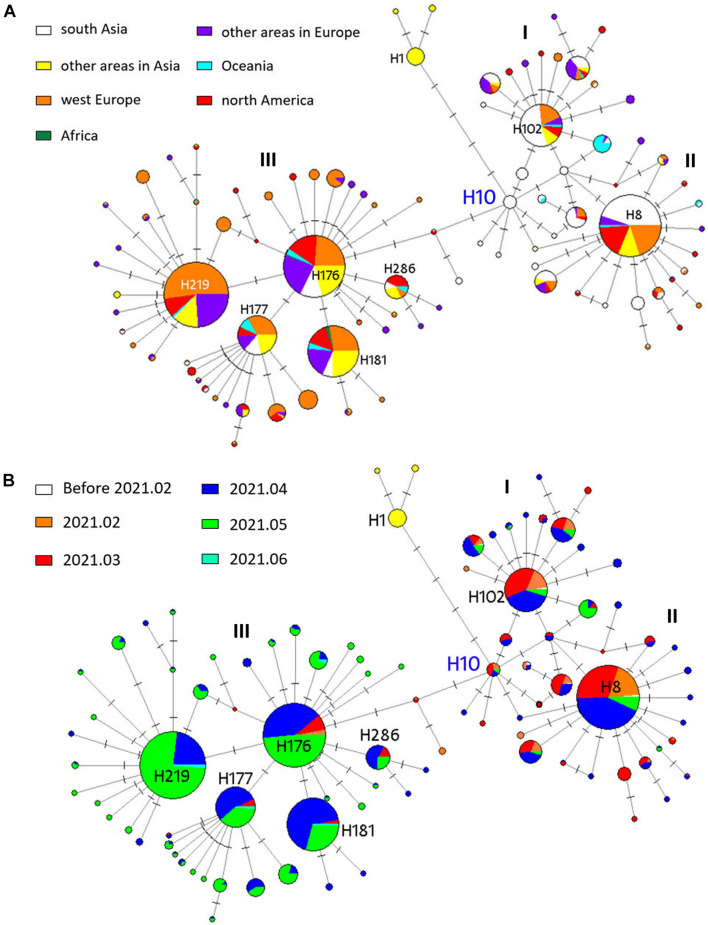
Phylogenetic network analysis of 1159 B.1.617 S genes. Circle areas in panel **(A)** are proportional to the number of geographical sites and in panel **(B)** are proportional to the time period. Each notch on the link represents a mutated nucleotide position. H10 was the root cluster of B.1.617 obtained with the early isolates in 2020.

To understand the evolution of B.1.617 sublineages in human hosts and trace infection pathways, we generated a phylogenetic network. Overall, the network showed that different newly mutated daughter nodes were derived from their ancestors (Figure 4). We used SARS-CoV-2 S encoding sequences from the early stage of the COVID-19 pandemic (prior to March 2020) as an outgroup (haplotype 1, H1), resulting in the root of the network being placed in H10. We found that the B.1.617 viruses and H1 were distinguished by four non-synonymous mutations: U1355G (L452R), G1450C (E484Q), A1841G (D614G), and C2042G (P681R). All the 10 sequences in the H10 were sampled in India. Three large individual nodes (I, II, and III) were evolved from H10, of which nodes I and II differed from H10 via the U333C (synonymous mutation), C3301G/T (H1101D) and U284C (I95T), and T333C mutations; while node III differed from H10 via the C56G (T19R), C1433A (T478K), and C1450G (Q484E) mutations.

Nodes I, II, and III corresponded to lineages B.1.617.3, B.1.617.1, and B.1.617.2, respectively. For B.1.617.3, H102 was the most prevalent haplotype, and 47 of the 87 sequences (54%) in H102 were sampled in India. H8 was the most prevalent B.1.617.1 haplotype, and 85 of the 190 sequences (45%) were sampled in India. With the pandemic ongoing, novel B.1.617.2 nodes were further derived from the ancestral node. B.1.617.2 is the most prevalent among the three sublineages; the number of individuals included in the nodal type and in mutational branches radiating was striking. Four major daughter network nodes or haplotypes (H219, H177, H181, and H286) were derived from B.1.617.2 and spread widely in different continents, especially around Europe (Figure 4A). B.1.617.1- and B.1.617.3-specific haplotypes were mainly prevalent before May 2021, while B.1.617.2 was prevalent since April (Figure 4B). In fact, 75.5% (16/184) of the B.1.617.2 H176 samples were collected in April and May, while 95.4% (83/87) of the B.1.617.3 H102 samples and 93.2% (177/190) of the B.1.617.1 H8 samples were identified prior to May.

### The Tertiary Structures of Receptor-Binding Domain and RNA-Dependent RNA Polymerase Mutants With Amino Acid Changes

**FIGURE 5:**
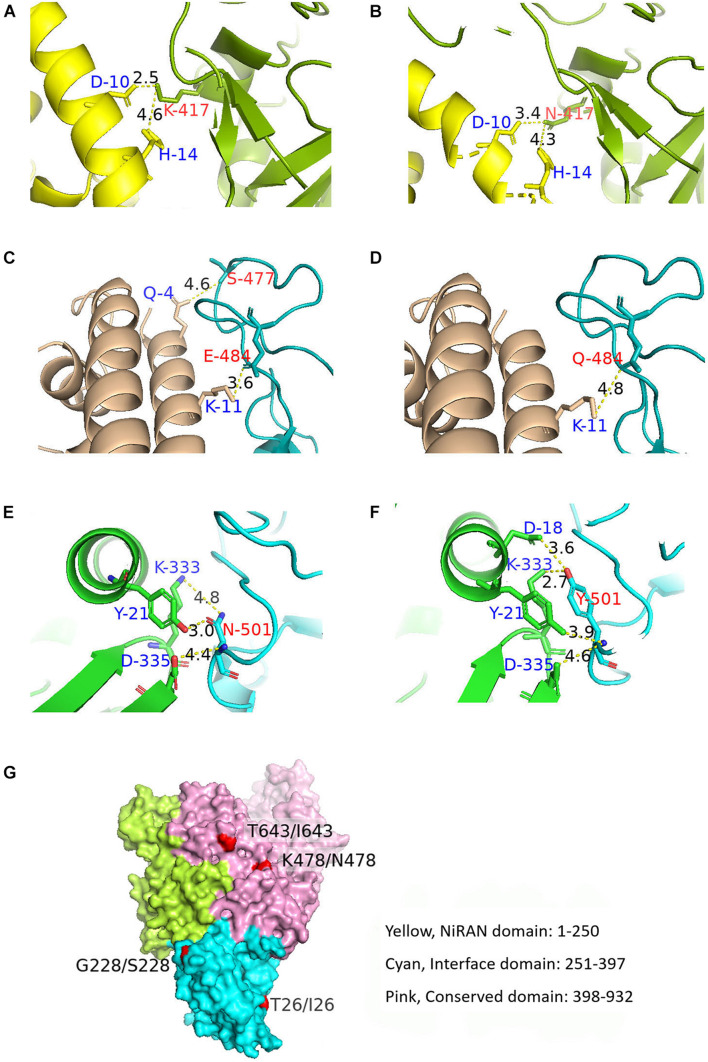
Docking structures showing the interaction between B.1.617 S-RBD and hACE2 and the tertiary B.1.617 RdRp structure. **(A,B)** Bonding networks of K417 and N417 aa residues showing no significant bonding network change in the two mutants. **(C,D)** Bonding networks of aa residues E484 and Q484 showing that the E484 mutation enhances the interaction with hACE2, compared with Q484. **(E,F)** Bonding network of aa residues N501 and Y501 showing that the Y501 mutation considerably enhances the interaction with hACE2, compared with N501. **(G)** RdRp spatial structure. The aa substitutions in RdRp did not cause spatial structural changes. Red: aa substitution sites on the protein surface. S-RBD, receptor-binding domain of the S protein; hACE2, human angiotensin-converting enzyme; RdRp, RNA-dependent RNA polymerase.

The aa substitutions with occurrence frequencies >1% were V382L, T478K, and E484Q in the RBD of the S protein (S-RBD). In addition, substitutions were also detected at the 417 (K to N) and 501 (N to Y) sites in the RBD, with low occurrence frequencies. Docking structures of B.1.617 wild-type S-RBD and the receptor [human angiotensin-converting enzyme (hACE2)] showed that residues 417, 484, and 501 have a bonding network with hACE2. Thus, corresponding three pairs (N417 and K417, E484 and Q484, and N501 and Y501) of docking structures between the S-RBD and hACE2 were analyzed. A low energy indicates a stable system of the interacted proteins, and we selected the structure with the lowest energy of each complex for further study. The docking energy score and root-mean-square deviation (RMSD) were calculated to evaluate the change in the structures of S-RBD; and the RBD–hACE2 complexes for K417, E484, and Y501 respectively revealed significant conformational changes to N417, Q484, and N501 (Table 2), and it was shown that the complexes of K417, E484, and Y501 were more stable than those of N417, Q484, and N501, respectively. Docking structures showed that residue 417 interacts with D10 and H14 in the hACE2 through two salt bridges. The K-to-N change at aa position 417 leads to the widening of the space between K417 and D10, which is compensated by the close proximity of K417 and H14 (Figures 5A,B). As a result, the K-to-N substitution at residue position 417 marginally affects the viral tertiary structure. Residue Q484 forms an interaction with K11 of hACE2 via hydrogen bonding at the distance of 4.8 Å. In the E484 mutant, the interaction between the two atoms is not only enhanced (3.6 Å) but a new bonding interaction (4.6 Å) forms between S477 in the S protein and Q4 of the hACE2 (Figures 5C,D). Thus, compared with Q484, E484 may serve to strengthen the interaction with hACE2. Furthermore, residue N501 of the S protein interacts with Y21, K333, and D335 of the hACE2, with bond lengths of 3.0, 4.8, and 4.4 Å, respectively. In the RBD–hACE2 complex of Y501, the interaction between Y501 and K333 is formed, with a much shorter bond length compared with N501 (2.7 Å) (Figure 5E). In addition, the Y501 substitution generated a new interaction between residues Y501 and D18 of hACE2 with a 3.6-Å bond length (Figures 5F,D). Due to the fact that the B.1.617 Y501 mutant, which originated from a US sample collected in April 2021, had a much stronger interaction with hACE2, it is reasonable for us to focus on monitoring its potential phenotypical changes.

**TABLE 2:** Docking scores for the S-RBD and hACE2 complexes.

**S-RBD mutants**	**K417**	**N417**	**E484**	**Q484**	**N501**	**Y501**
Docking score (kcal/mol)	–360.62	–329.41	–360.62	–339.61	–308.98	–354.57
Ligand RMSD (Å)	0.41	0.49	0.41	0.45	0.96	0.43

*RMSD, root-mean-square deviation; S-RBD, receptor-binding domain of the S protein; hACE2, human angiotensin-converting enzyme.*

Besides the five aa substitutions (T26I, K478N, T643I, G671S, and V675) described above with occurrence frequencies >1% in the RdRp, there were other two sites (G228S and I244V) with frequencies <1% but detected in more than four sequences. Of the seven aa substitutions targeting RdRp, three were located in the nidovirus RdRp-associated nucleotidyltransferase (NiRAN) domain, and the other four were located in the conserved polymerase domain (Figure 5G). No aa substitution sites were identified in the interface domain (Figure 5G). Tertiary structure analysis showed that the aa sites 26, 228, 478, and 643 were located on the protein surface and that aa substitutions at these four sites did not alter the spatial structure of RdRp (Figure 5G).

## Discussion

Since the discovery of the D641G variant in early 2020, the continuous emergence of SARS-CoV-2 variants has attracted increasing attention worldwide. B.1.617 was first reported in India at the end of 2020 and has spread rapidly and globally, becoming the most prevalent SARS-CoV-2 variant to date. As analysis of genetic variability and evolution is necessary for the characterization of viral variants and the investigation of how they impact disease severity and transmissibility, and to what extent they affect the performance of diagnostic tests, vaccines, social measures etc., the dynamic genetic features on B.1.617 SARS-CoV-2 genomes were analyzed and evaluated in this study.

In sharp contrast to the small number of B.1.617 sequences and their limited geographical distribution in early 2021, both parameters have dramatically increased since March 2021, reflecting the rapid global spread of B.1.617. Mutation, a phenomenon that naturally occurs over time, is an important factor for viral evolution, providing the virus with opportunities to gain fitness (leading to enhanced transmission and/or improved immune evasion) in the infected host. SNPs, shown to be widely distributed across the B.1.617 genomes (and especially those with high occurring frequencies), are more commonly present in structural rather than non-structural protein genes. Besides site-specific mutations, deletions are found in the S, ORF8, and nsp1 sequences, which may potentially influence the function of proteins participating in viral infection, transmission, and immunomodulation (Islam et al., 2020; Phan, 2020; Zhou et al., 2020). As mentioned above in the section of introduction, some mutations were identified to be related to the altered phenotype of B.1.617 variants, but many problems remained to be solved. For instance, do the E156Δ/F157Δ aa deletions in the S protein arm the VOC B.1.617.2 variant with improved transmissibility or reduce its susceptibility to the preexisting neutralizing antibody? This needs to be determined by further *in vivo* studies.

Methodologically, it is of great importance to infer function from the conservation degree of the protein as a whole or even the individual aa. Positive or diversifying selection can accelerate the fixation of advantageous non-synonymous mutations, while negative or purifying selection can avoid deleterious mutations and favor the optimization of viral features (Daugherty and Malik, 2012). We found evidences of both diversifying and purifying selection in different B.1.617 variant proteins, with the former selection type dominant in structural proteins (especially the S protein) and the latter one dominant in non-structural proteins (such as nsp3 and nsp12). Generally, the presence of diversifying selection may suggest an impact of the host immune response on the variants, whereby viruses could escape from the protective immunity of the host, especially for the neutralizing antibodies, during the course of the pandemic. In fact, reduced neutralization of B.1.617.1 and B.1.617.2 variants by antibodies or convalescent sera elicited as a result of vaccination or nature infection has been reported (Hu et al., 2021; Planas et al., 2021; Supasa et al., 2021). A subset of positively selected sites were found in different proteins of SARS-CoV-2 (Berrio et al., 2020; Velazquez-Salinas et al., 2020; Emam et al., 2021; Rochman et al., 2021), which are informative to evaluate their impacts on the protein structure and the processes critical to the viral life cycle, including replication, transcription, translation, and RNA stability. Purifying selection indicates the importance of steadying the vital functional proteins or domains associated with major functions such as viral replication and infection. Thus, a large number of purifying selection mutations detected in the nsp3 suggest a highly conserved pattern in its function in viral genome replication and in antagonizing the host innate immunity (Dong et al., 2020). Generally, viruses tread a tightrope between low pathogenicity and high infectivity, which is beneficial to their long-term survival. However, statistical data from Scotland and England seemingly exhibited an increased risk of hospitalization among individuals infected with the B.1.617.2 variant, compared with those infected with the early variant B.1.617.1 (PublicHealthEngland, 2021). Regarding viral transmissibility, it was also reported that the estimated effective reproductive number of B.1.617.2 was 55% higher than that of the earlier variants (B.1.1.7, B.1.351, and P.1) and 97% (95% CI: 76–117) higher than that of other non-VOC/VOI members (Campbell et al., 2021). Of course, it is necessary to stress that all the evaluations on viral property need to be verified carefully by experiments.

Phylogenetic analysis showed no obvious spatial–temporal specificity for B.1.617.3 due to a relatively low epidemic intensity, B.1.617.1 was prevalent in the early stages of the pandemic and mainly localized to Southern Asia, and B.1.617.2 was predominant since April 2021 and assumed a global distribution. The haplotype network in this study also demonstrated the same result as the phylogenetic analysis. Although we cannot rule out the potential for sampling bias and mutagenesis bias, which could have led to inaccuracies in this study, our results imply that B.1.617.2 viruses rapidly spread through the population. Furthermore, haplotype network analysis has been applied to SARS-CoV-2 data to understand the viral evolution and trace the infection pathways (Forster et al., 2020a,b). Our network showed that India was the epicenter for B.1.617 variants and that three major nodes were generated from the original haplotype. Furthermore, B.1.617.1 and B.1.617.3 each contained a widely prevalent haplotype. Meanwhile, four additional daughter haplotypes with high prevalence were derived from the prevalent ancestral B.1.617.2 haplotype (H176). Numbers of different haplotypes that were detected in the B.1.617 lineage revealed that a large number of mutations were generated during the process of viral infection and transmission. Some of these novel variants (e.g., B.1.617.2 H219 and H177) still exhibited a good fitness and were widely prevalent in the population. It is worth noting that the phylogeographic patterns in the network are potentially affected by many factors, such as founder effects and sample size (Forster et al., 2020a).

The S1 subunit in the N-terminal region of the S protein contains the RBD (aa 319–541), of which aa 437–508 constitute the receptor-binding motif (Winger and Caspari, 2021), and 17 amino acids make direct contact with hACE2: (K417, G446, Y449, Y453, L455, F456, A475, F486, N487, Y489, Q493, G496, Q498, T500, N501, G502, and Y505) (Lan et al., 2020). The aa substitutions in the S-RBD may cause structural and functional changes. Typically, computational or bioinformatics tools can be applied to investigate tertiary structure and the resulting host–pathogen interactions (Bakhshandeh et al., 2021). The K-to-N substitution at aa site 417 in the S protein did not cause significant changes to the surface interaction between RBD and hACE2. The substitution of E to Q at aa site 484 lengthened the interaction distance between the original residues (Q484 of RBD and K11 of hACE2) and reduced the interaction between the two other residues (S477 of RBD and Q4 of hACE2), which may have weakened the affinity between the S protein and its receptor and may function in antibody escape (Planas et al., 2021). However, functional experiments are needed to verify these speculations. N501Y substitution was detected in B.1.1.7 and B.1.351, and it was suggested to be responsible for the enhanced replication in cell and in animal model and for the potentially increased transmission (Liu et al., 2021c). In the present study, two B.1.617 sequences from the United States were found to contain the N501Y substitution. Tertiary structure analysis showed that compared with N501, Y501 had a much stronger interaction with hACE2. Notably, during the preparation of this manuscript, we continued to analyze the sequences entered into GISAID throughout June and July 2021 and found that the Y501 mutant was also detected in Turkey, Poland, Czech Republic, Germany, and Cambodia, reminding us of the urgent need for the close surveillance of this variant. However, a causal link between the increased binding to hACE2 and the elevated replication in host cells cannot be drawn currently. For an enveloped virus, besides attachment to the receptor, those other factors, such as the cleavage efficiency by furin, that affect membrane fusion are critically important for entry into the cells lining the respiratory tract and the ensuing replication.

Despite the presence of seven aa substitutions, no obvious structural changes were observed in the RdRp. Combined with the fact that no aa changes occurs in nsp7 or nsp8, the two important proteins serving to promote RdRp-mediated replication (Posthuma et al., 2017; Biswal et al., 2021), it was concluded that the replication activity of RdRp of B.1.617 remained steady.

In conclusion, the data reported in this study showed that widely distributed SNPs and aa substitutions were detected across the B.1.617 viral genomes, especially in the S protein. Twenty-two positively selected sites were detected and 16 were non-C-to-U transitions. The aa substitution of N501Y can potentially enhance the interactions between S-RBD and ACE2, and the variants bearing N501Y were increasingly distributed in more countries in the follow-up monitoring. Therefore, we have improved our knowledge about the genetic diversity of B.1.617 lineage and the potential impact on viral property individually and as a whole. The biological significance and the underlining evolution rule of these variants merit further attention and verification.

## Data Availability Statement

The datasets presented in this study can be found in online repositories. The names of the repository/repositories and accession number(s) can be found in the article/Supplementary Material.

## Author Contributions

J-mY and J-sH designed the study. L-qF, X-yH, Y-yC, and X-lP collected the data. J-mY, L-qF, X-yH, Y-yC, Y-hF, and Y-pZ analyzed the data. J-mY, L-qF, X-yH, and Y-yC composed the manuscript. J-sH reviewed the manuscript. All authors have read and approved the manuscript.

## Conflict of Interest

The authors declare that the research was conducted in the absence of any commercial or financial relationships that could be construed as a potential conflict of interest.

## Publisher’s Note

All claims expressed in this article are solely those of the authors and do not necessarily represent those of their affiliated organizations, or those of the publisher, the editors and the reviewers. Any product that may be evaluated in this article, or claim that may be made by its manufacturer, is not guaranteed or endorsed by the publisher.

## Footnotes

1
https://covid19.who.int

2
https://www.gisaid.org

3
https://www.ncbi.nlm.nih.gov/genbank

4
https://mafft.cbrc.jp/alignement/server/

5
https://www.viprbrc.org/brc/

6
http://weblogo.berkeley.edu/logo.cgi

7
http://www.datamonkey.org/

8
http://www.sbg.bio.ic.ac.uk/phyre2

9
http://hdock.phys.hust.edu.cn/

## References

[B1] Bakhshandeh B. Jahanafrooz Z. Abbasi A. Goli M. B. Sadeghi M. Mottaqi M. S. (2021). Mutations in SARS-CoV-2; consequences in structure, function, and pathogenicity of the virus. *Microb. Pathog.* 154:104831. 10.1016/j.micpath.2021.104831 33727169 PMC7955574

[B2] Berrio A. Gartner V. Wray G. A. (2020). Positive selection within the genomes of SARS-CoV-2 and other Coronaviruses independent of impact on protein function. *PeerJ* 8:e10234. 10.7717/peerj.10234 33088633 PMC7571416

[B3] Biswal M. Diggs S. Xu D. Khudaverdyan N. Lu J. Fang J. (2021). Two conserved oligomer interfaces of NSP7 and NSP8 underpin the dynamic assembly of SARS-CoV-2 RdRP. *Nucleic Acids Res.* 49 5956–5966. 10.1093/nar/gkab3706276908 33999154 PMC8191759

[B4] Campbell F. Archer B. Laurenson-Schafer H. Jinnai Y. Konings F. Batra N. (2021). Increased transmissibility and global spread of SARS-CoV-2 variants of concern as at June 2021. *Euro Surveill.* 26:2100509. 10.2807/1560-7917.ES.2021.26.24.2100509 34142653 PMC8212592

[B5] Daugherty M. D. Malik H. S. (2012). Rules of engagement: molecular insights from host-virus arms races. *Annu. Rev. Genet.* 46 677–700. 10.1146/annurev-genet-110711-155522 23145935

[B6] Deng X. Garcia-Knight M. A. Khalid M. M. Servellita V. Wang C. Morris M. K. (2021). Transmission, infectivity, and neutralization of a spike L452R SARS-CoV-2 variant. *Cell* 184 3426–3437.e8. 10.1016/j.cell.2021.04.025 33991487 PMC8057738

[B7] Dong S. Sun J. Mao Z. Wang L. Lu Y. L. Li J. (2020). A guideline for homology modeling of the proteins from newly discovered betacoronavirus, 2019 novel coronavirus (2019-nCoV). *J. Med. Virol.* 92 1542–1548. 10.1002/jmv.25768 32181901 PMC7228330

[B8] Emam M. Oweda M. Antunes A. El-Hadidi M. (2021). Positive selection as a key player for SARS-CoV-2 pathogenicity: insights into ORF1ab, S and E genes. *Virus Res.* 302:198472. 10.1016/j.virusres.2021.198472 34118359 PMC8190378

[B9] Forster P. Forster L. Renfrew C. Forster M. (2020a). Phylogenetic network analysis of SARS-CoV-2 genomes. *Proc. Natl. Acad. Sci. U. S. A.* 117 9241–9243. 10.1073/pnas.2004999117 32269081 PMC7196762

[B10] Forster P. Forster L. Renfrew C. Forster M. (2020b). Reply to Sanchez-Pacheco et al., Chookajorn, and Mavian et al.: explaining phylogenetic network analysis of SARS-CoV-2 genomes. *Proc. Natl. Acad. Sci. U. S. A.* 117 12524–12525. 10.1073/pnas.2007433117 32439706 PMC7293629

[B11] Hu J. Wei X. Y. Xiang J. Peng P. Xu F. L. Wu K. (2021). Reduced neutralization of SARS-CoV-2 B.1.617 variant by inactivated and RBD-subunit vaccine. *bioRXiv* [Preprint] 10.1101/2021.07.09.451732

[B12] Islam M. R. Hoque M. N. Rahman M. S. Alam A. Akther M. Puspo J. A. (2020). Genome-wide analysis of SARS-CoV-2 virus strains circulating worldwide implicates heterogeneity. *Sci. Rep.* 10:14004. 10.1038/s41598-020-70812-6 32814791 PMC7438523

[B13] Lan J. Ge J. Yu J. Shan S. Zhou H. Fan S. (2020). Structure of the SARS-CoV-2 spike receptor-binding domain bound to the ACE2 receptor. *Nature* 581 215–220. 10.1038/s41586-020-2180-5 32225176

[B14] Liu Y. Liu J. Johnson B. A. Xia H. Ku Z. Schindewolf C. (2021b). Delta spike P681R mutation enhances SARS-CoV-2 fitness over Alpha variant. *bioRxiv* [Preprint] 10.1101/2021.08.12.456173 35550680 PMC9050581

[B15] Liu C. Ginn H. M. Dejnirattisai W. Supasa P. Wang B. Tuekprakhon A. (2021a). Reduced neutralization of SARS-CoV-2 B.1.617 by vaccine and convalescent serum. *Cell* 184 4220–4236.e13. 10.1016/j.cell.2021.06.020 34242578 PMC8218332

[B16] Liu Y. Liu J. Plante K. S. Plante J. A. Xie X. Zhang X. (2021c). The N501Y spike substitution enhances SARS-CoV-2 transmission. *bioRXiv* [Preprint] 10.1101/2021.03.08.434499 34818667 PMC8900207

[B17] Matyasek R. Kovarik A. (2020). Mutation patterns of human SARS-CoV-2 and bat RaTG13 coronavirus genomes are strongly biased towards C>U Transitions, indicating rapid evolution in their hosts. *Genes (Basel)* 11:761. 10.3390/genes11070761 32646049 PMC7397057

[B18] Peacock T. P. Sheppard C. M. Brown J. C. Goonawardane N. Zhou J. Whiteley M. (2021). The SARS-CoV-2 variants associated with infections in India, B.1.617, show enhanced spike cleavage by furin. *bioRXiv* [Preprint]. 10.1101/2021.05.28.446163

[B19] Phan T. (2020). Genetic diversity and evolution of SARS-CoV-2. *Infect. Genet. Evol.* 81:104260. 10.1016/j.meegid.2020.104260 32092483 PMC7106203

[B20] Planas D. Veyer D. Baidaliuk A. Staropoli I. Guivel-Benhassine F. Rajah M. M. (2021). Reduced sensitivity of SARS-CoV-2 variant Delta to antibody neutralization. *Nature* 596 276–280. 10.1038/s41586-021-03777-9 34237773

[B21] Pond S. L. Murrell B. Poon A. F. (2012). Evolution of viral genomes: interplay between selection, recombination, and other forces. *Methods Mol. Biol.* 856 239–272. 10.1007/978-1-61779-585-5_10 22399462

[B22] Posthuma C. C. Te Velthuis A. J. W. Snijder E. J. (2017). Nidovirus RNA polymerases: complex enzymes handling exceptional RNA genomes. *Virus Res.* 234 58–73. 10.1016/j.virusres.2017.01.023 28174054 PMC7114556

[B23] PublicHealthEngland (2021). *SARS-CoV-2 Variants of Concern and Variants Under Investigation in England - Technical briefing 15.* London: PHE.

[B24] Rochman N. D. Wolf Y. I. Faure G. Mutz P. Zhang F. Koonin E. V. (2021). Ongoing global and regional adaptive evolution of SARS-CoV-2. *Proc. Natl. Acad. Sci. U. S. A.* 118:e2104241118. 10.1073/pnas.2104241118 34292871 PMC8307621

[B25] Saito A. Nasser H. Uriu K. Kosugi Y. Irie T. Shirakawa K. (2021). SARS-CoV-2 spike P681R mutation enhances and accelerates viral fusion. *bioRXiv* [Preprint] 10.1101/2021.06.17.448820

[B26] Spielman S. J. Weaver S. Shank S. D. Magalis B. R. Li M. Kosakovsky Pond S. L. (2019). Evolution of viral genomes: interplay between selection, recombination, and other forces. *Methods Mol. Biol.* 1910 427–468. 10.1007/978-1-4939-9074-0_14 31278673

[B27] Su S. Wong G. Shi W. Liu J. Lai A. C. K. Zhou J. (2016). Epidemiology, genetic recombination, and pathogenesis of coronaviruses. *Trends Microbiol.* 24 490–502. 10.1016/j.tim.2016.03.003 27012512 PMC7125511

[B28] Supasa P. Zhou D. Dejnirattisai W. Liu C. Mentzer A. J. Ginn H. M. (2021). Reduced neutralization of SARS-CoV-2 B.1.1.7 variant by convalescent and vaccine sera. *Cell* 184 2201–2211.e7. 10.1016/j.cell.2021.02.033 33743891 PMC7891044

[B29] van Dorp L. Richard D. Tan C. C. S. Shaw L. P. Acman M. Balloux F. (2020). No evidence for increased transmissibility from recurrent mutations in SARS-CoV-2. *Nat. Commun.* 11:5986. 10.1038/s41467-020-19818-2 33239633 PMC7688939

[B30] Velazquez-Salinas L. Zarate S. Eberl S. Gladue D. P. Novella I. Borca M. V. (2020). Positive selection of ORF1ab, ORF3a, and ORF8 genes drives the early evolutionary trends of SARS-CoV-2 during the 2020 COVID-19 pandemic. *Front. Microbiol.* 11:550674. 10.3389/fmicb.2020.550674 33193132 PMC7644918

[B31] Winger A. Caspari T. (2021). The spike of concern-the novel variants of SARS-CoV-2. *Viruses* 13:1002. 10.3390/v13061002 PMC8229995 34071984

[B32] Yadav P. D. Mohandas S. Shete A. M. NYayanit D. A. Gupta N. Patil D. Y. (2021a). SARS CoV-2 variant B.1.617.1 is highly pathogenic in hamsters than B.1 variant. *bioRxiv* [Preprint] 10.1101/2021.05.05.442760

[B33] Yadav P. D. Sapkal G. N. Abraham P. Ella R. Deshpande G. Patil D. Y. (2021b). Neutralization of variant under investigation B.1.617 with sera of BBV152 vaccinees. *Clin. Infect. Dis.* ciab411. 10.1093/cid/ciab411 [Online ahead of print]. 33961693

[B34] Zhang J. Nielsen R. Yang Z. (2005). Evaluation of an improved branch-site likelihood method for detecting positive selection at the molecular level. *Mol. Biol. Evol.* 22 2472–2479. 10.1093/molbev/msi237 16107592

[B35] Zhao Z. Li H. Wu X. Zhong Y. Zhang K. Zhang Y. P. (2004). Moderate mutation rate in the SARS coronavirus genome and its implications. *BMC Evol. Biol.* 4:21. 10.1186/1471-2148-4-21 15222897 PMC446188

[B36] Zhou Y. Hou Y. Shen J. Huang Y. Martin W. Cheng F. (2020). Network-based drug repurposing for novel coronavirus 2019-nCoV/SARS-CoV-2. *Cell Discov.* 6:14. 10.1038/s41421-020-0153-3 PMC7073332 32194980

